# An alternatively spliced variant of CXCR3 mediates the metastasis of CD133+ liver cancer cells induced by CXCL9

**DOI:** 10.18632/oncotarget.7360

**Published:** 2016-02-13

**Authors:** Qiang Ding, Yujia Xia, Shuping Ding, Panpan Lu, Liang Sun, Mei Liu

**Affiliations:** ^1^ Department of Gastroenterology, Tongji Hospital, Tongji Medical College, Huazhong University of Science and Technology, Wuhan, Hubei Province, China; ^2^ Department of Gastroenterology, The Central Hospital of Wuhan, Tongji Medical College, Huazhong University of Science and Technology, Wuhan, Hubei Province, China

**Keywords:** CXCR3/CXCL9, CD133+ liver cancer cells, metastasis, adhesion

## Abstract

Metastasis of liver cancer is closely linked to tumor microenvironment, in which chemokines and their receptors act in an important role. The CXCR3, the receptor of chemokine CXCL9, belongs to a superfamily of rhodopsin-like seven transmembrane GPCRs and CXCR subfamily. In HCC tissues, CXCR3 was frequently upregulated and correlated with tumor size, tumor differentiation, portal invasion and metastasis. In the study, CXCR3-A isoform that was bound by CXCL9 was found to cause significant change of ERK1/2 phosphorylation level in the MAPK signaling pathway, consequently upregulating the MMP2 and MMP9 expression and promoting invasion and metastasis of CD133+ liver cancer cells. Also, CXCR3-A suppressed the adhesion ability of CD133+ liver cancer cells that stimulated by CXCL9 for 24h. These findings suggest that CXCR3 and its ligand CXCL9 could promote the metastasis of liver cancer cells and might be a potential target for the intervention of liver cancer metastasis.

## INTRODUCTION

Hepatocellular carcinoma (HCC) is the fifth most common cancer, and it is also the third most common cancer leading to the death worldwide [1]. Chronic HBV infection is the most prominent risk factor contributing to HCC [2]. Although various surgical and medical interventions become available, the prognosis of HCC patients is still poor due to the lack of reliable early-stage screening tests, metastasis, and a high rate postsurgical recurrence [3]. Therefore, the fundamental understanding of the molecular pathogenesis of HCC recurrence and metastasis is critical for the improvement of HCC therapies.

Tumor metastasis is a multistage process during which the cancer cells separate from the primary tumor, survive in the circulation, seed at distant sites, and grow [4]. Emerging evidence indicates that tumor microenvironment is significant in metastasis and invasion of tumor cells, in which chemokines are a vital constituent part [5]. Chemokines are small secreted proteins (8–12 kDa) that share structural similarities, and the members of this molecular superfamily can be subdivided into four classes, the C-C, C-X-C, C and C-X3-C chemokines, depending on the location of the first two conserved N-terminal cysteine residues that build disulfide bonds to two other cysteine residues within the protein sequence [6]. It is commonly believed that HBV is the major etiological cause of HCC [7]. Previous research in our laboratory suggested that HBV protein X (HBx) can activate nuclear factor-kappa B (NF-kB), subsequently upregulating the chemokine CXCL9 expression [8]. CXCL9 belongs to the CXC chemokines family, which is involved in some inflammatory diseases such as rheumatoid arthritis [9], recruitment of T cells [10], and angiogenesis [11]. CXCR3, a G-protein coupled receptor of chemokine CXCL9, is significant in tumor progression and angiogenesis, and also has been found to be correlated with poor prognosis for breast tumors, melanoma, and colon and renal cancer patients [12]. Three splice variants for CXCR3 have been established as CXCR3-A, CXCR3-B and CXCR3-alt. The CXCR3-alt isoform is still able to mediate CXCL11- but not CXCL9- or CXCL10-dependent activity [13]. However, the role and molecular mechanism of chemokine CXCL9 with CXCR3 receptor isoforms in the metastasis and development of HCC has not been reported.

Cancer metastasis is facilitated by the remodeling of the extracellular matrix (ECM) by a family of proteolytic enzymes known as the matrix metalloproteinases (MMPs). MMPs encompass 24 related enzymes and are subdivided into categories depending on their substrate specificity [14]. Among the enzymes, MMP2 and MMP9 have been extensively studied for different cancers, such as gastric cancer, prostate cancer, gynecological cancer, and brain cancer [15–18]. Their activation could be regulated by the MAPK signaling pathway [19, 20]. In this research, we would like to investigate the likely underlying relationship among MMPs, MAPK family, and chemokine CXCL9/CXCR3 isform.

Cancer stem cells have become a hotspot in the study of carcinogenesis. Considerable research with liver cancer stem cells have been investigated recently, and CD133 was found to be an important liver cancer stem cell mark [21, 22]. Considerable evidence showed that chemokines and its receptors are closely related with some biological properties of cancer stem cells [23, 24], so our research team wondered whether the CXCL9 and CXCR3 receptors are able to affect the CD133+ liver cancer cells, and isolates CD133+ cells from Huh7, Hep-3B, and PLC/PRF/5 cell lines to start further research.

In summary, chemokine CXCL9 was found to facilitate the metastasis ability of HCC CD133+ cancer cells, and CXCR3 receptor subtype CXCR3-A was found to play a key role during the metastasis, which was studied in this current study. In addition, CXCL9/CXCR3-A was also confirmed to promote the ability of metastasis by activating the ERK1/2-MMP2/MMP9 pathway.

## RESULTS

### Expression of CXCR3 in HCC tissues

**Figure 1:**
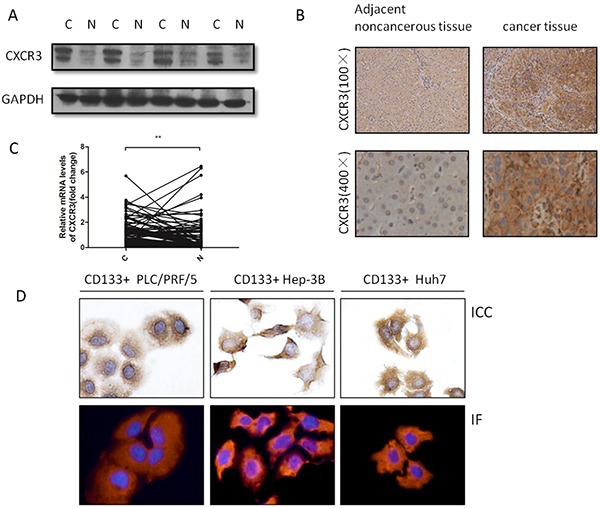
CXCR3 is upregulated in HCC tissues and also expressed in CD133+ liver cancer cells **A.** Representative image showed CXCR3 expression in four pairs HCC tissues and the adjacent noncancerous tissues. **B.** Representative immunohistochemical staining showed the expression of CXCR3 in HCC tissues and the adjacent noncancerous tissues. CXCR3 was present in the cytoplasm and membrane, but not in the cell nucleus. **C.** The expressions of CXCR3 mRNA in 89 pairs HCC and the corresponding pericarcinoma tissues were measured by real-time RT-PCR. **P < 0.01. Data are represented as the mean ± SD. **D.** ICC and IF identify the expression of CXCR3 in CD133+ PLC/PRF/5, Hep-3B and Huh7 cells. The ICC and IF results showed that CXCR3 was present in the cytoplasm and membrane C, liver cancer tissues; N, liver adjacent noncancerous tissues

Immunohistochemistry was performed to explore the expression of the CXCR3 receptor in HCC tissues. The results showed that CXCR3 was predominately expressed in cell membrane and cytoplasm, and was upregulated significantly in cancer tissues in comparison with the corresponding adjacent noncancerous tissues(27/40, 67.5%) (Figure 1B). Also, mRNA level of CXCR3 were examined, the result showed that CXCR3 expression was predominately higher in cancer tissues than the corresponding adjacent noncancerous tissues (63/89, 70.79%) (Figure 1C). Further analysis of the clinicopathologiacal characteristics in 89 paired HCC tissues showed CXCR3 expression was positively correlated with the tumor side(p=0.014), tumor differentiation(p=0.014), portal invasion(p=0.028), and metastasis(p=0.017) (Table 1). In addition, it was found that CXCR3 was expressed significantly in cancer tissues (29/40, 72.5%) through employing the western blot (Figure 1A).

**Table 1:** Clinical data and analysis of 89 pairs of HCC specimens

Variable	CXCR3			p value
	High expression^A^ (n = 63)	Not high expression (n = 26)	Percentage of high expression (70.79 %)	
Sex				
Male	53	21	71.6	0.759
Female	10	5	66.7	
Age(year)				
≤50	41	12	77.4	0.153
>50	22	14	61.1	
Tumor diameter (cm)				
≤5	16	14	53.3	0.014*
>5	47	12	79.7	
Tumor Number				
Single	51	23	68.9	0.538
Multi	12	3	80.0	
Tumor differentiation				
Poorly or moderately	56	17	76.7	0.014*
Well	7	9	43.8	
Portal invasion				
Absent	44	24	64.7	0.028*
Present	19	2	82.6	
HBsAg				
Negative	1	1	50.0	0.501
Positive	62	25	71.3	
AFP (ng/ml)				
≤400	32	15	68.1	0.643
>400	31	11	73.8	
Metastasis				
Yes	43	10	81.1	0.017*
No	20	16	55.6	

A mRNA levels of CXCR3 in the hepatocellular carcinoma tissue are higher than those in corresponding adjacent noncancerous tissue.

* indicates statistical significance.

### ICC and IF identify the CXCR3 receptor of CD133+ HCC cell lines

The CD133+ liver cancer cells were isolated from the PLC/PRF/5, Hep-3B and Huh7 cell lines by fluorescence-activated cell sorting (FACS). First, immunocytochemistry (ICC) and immunofluorescence (IF) were employed to prove the existence of the CXCR3 receptor (Figure 1D).

### Chemokine CXCL9 promotes the invasion and migration capability of CD133+ Huh7, Hep-3B and PLC/PRF/5 cells

**Figure 2:**
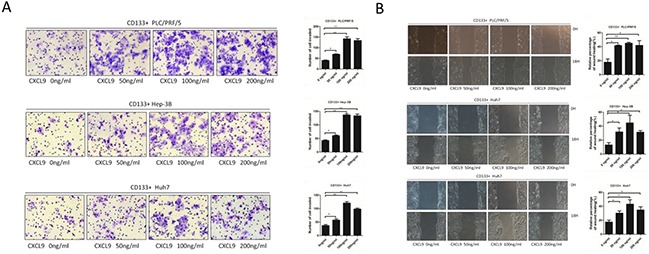
CXCL9 promote the migration and invasion ability of CD133+ PLC/PRF/5, Hep-3B and Huh7 cells **A.** Transwell assays showed that CXCL9 significantly facilitated the invasion and migration abilty of CD133+ PLC/PRF/5, Hep-3B and Huh7 cells. The numbers of invasive and migratory cells were quantified. **B.** Wounding healing tests showed that CXCL9 pomoted the migration ability of CD133+ PLC/PRF/5, Hep-3B and Huh7 cells. Cell migration was quantified as percentage of wound-healed area. **P < 0.01,*P < 0.05. Data are represented as mean +/− SEM.

To identify whether CXCL9 is able to enhance the cell invasion and migration, chemokine CXCL9 at different concentrations was added into the lower transwell chamber, ranging from 0 ng/ml to 200 ng/ml, and CD133+ liver cancer cells was added into the upper chamber. The portion of CD133+ Huh7, Hep-3B and PLC/PRF/5 cells migrate to the lower chamber in the experimental group were larger than those in the control group, which 100 ng/ml group reached the highest (Figure 2A).

Moreover, in order to determine the impact of CXCL9 on the mobility of CD133+ liver cancer cells, the wound healing assay was performed. Consistent with the previous observations, the groups with CXCL9 added showed more enhanced mobility than the control group, and the 100 ng/ml groups were also the highest (Figure 2B).

### CXCR3-A enhances invasion and migration of CD133+ liver cancer cells in vitro, and promotes hepatic metastasis in vivo

**Figure 3:**
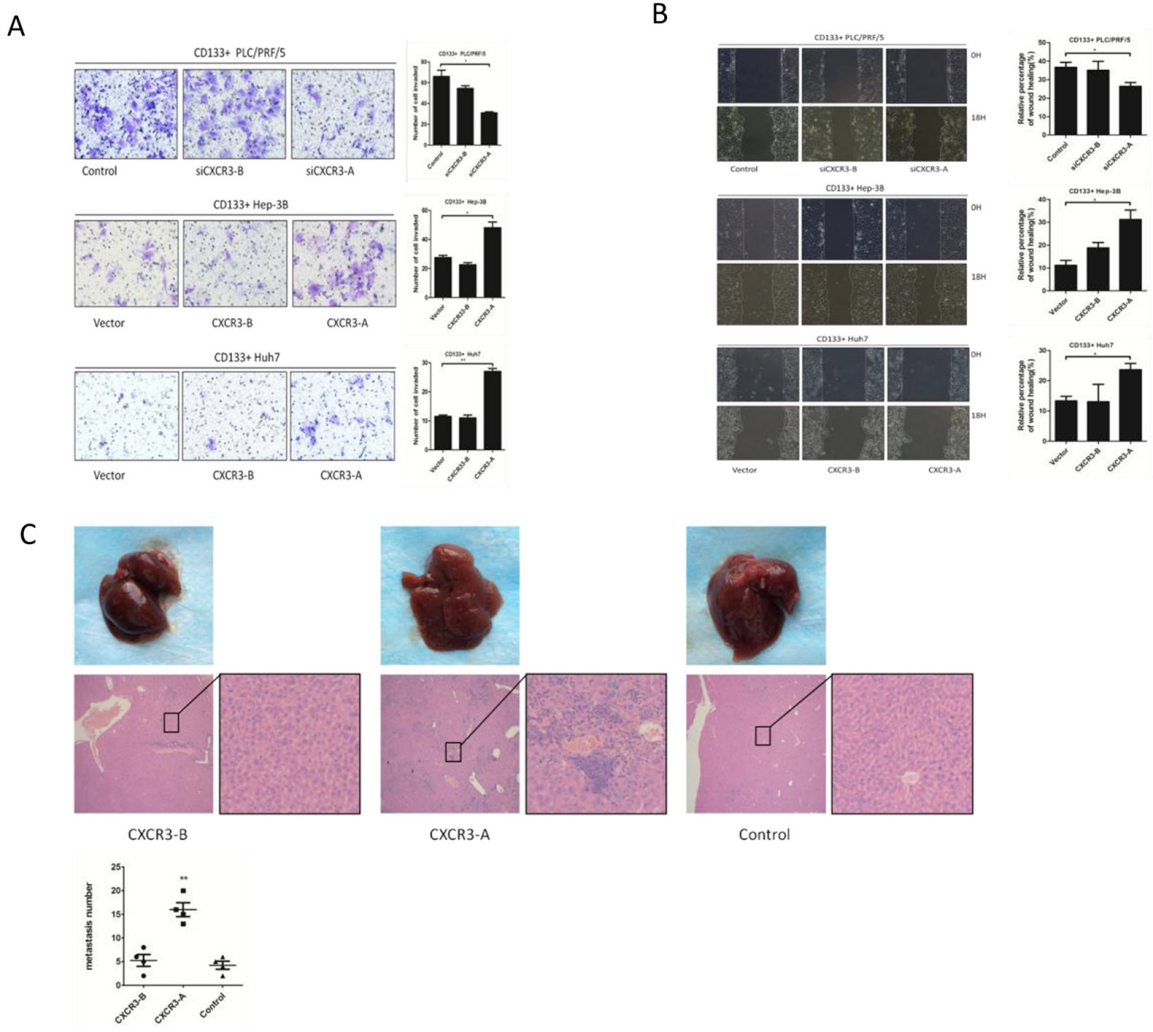
CXCR3-A enhances invasion and migration of CD133+ liver cancer cells in vitro, and promotes hepatic metastasis in vivo **A.** Transwell assays show the invasion and migration ability of CXCR3-A or CXCR3-B siRNA in CD133+ PLC/PRF/5 cells, CXCR3-A or CXCR3-B overexpression in CD133+ Hep-3B and Huh7 cells. **B.** CXCR3-A or CXCR3-B siRNA in CD133+ PLC/PRF/5 cells, CXCR3-A or CXCR3-B overexpression in CD133+ Hep-3B and Huh7 cells were performed by wounding healing test. *p<0.05. **C.** Representative liver metastasis images and their H&E staining images were showed (n = 4 per group). **P < 0.01. Data are represented as mean +/− SEM.

CXCR3 has three isoforms, but CXCL9 can only be bound to CXCR3-A and CXCR3-B [13]. To identify which CXCR3 receptor isoform determines the CXCL9 induced enhancement of invasion and migration, we employed the transwell assay and wound healing assay, and found the promotion of invasion and migration to be dependent on the CXCR3-A receptor isoform. The CD133+ Huh7 and Hep-3B with stable expression of CXCR3-A exhibited enhanced invasion and migration compared to the controls and the CXCR3-B groups in transwell assays and wound healing tests (Figure 3A and 3B). The RNA interference (RNAi) was used to knockdown the CXCR3-A or CXCR3-B expression of CD133+ PLC/PRF/5 cells, the tests showed the cells in which CXCR3-A expression were knockdown exhibited lower invasion and migration ability. Having observed that CXCR3-A promoted the CD133+ liver cancer cells migration in vitro, the pro-metastasis function of CXCR3-A was further confirmed in vivo by animal models. The CD133+ Hep-3B cells stably expressing CXCR3-A, CXCR3-B and Vector were injected into the lateral tail vein of mice. Consistent with the results in vitro, mice injected with CD133+ Hep-3B-CXCR3-A cells showed obvious liver metastasis, as compared to the CXCR3-B and control mice (Figure 3C).

### CXCR3-A suppresses cell adhesion in response to CXCL9

**Figure 4:**
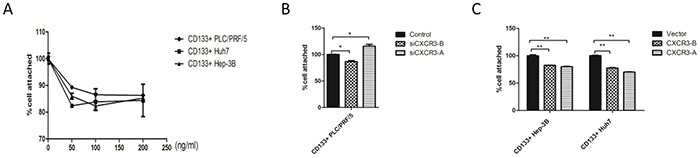
CXCR3-A suppresses cell adhesion in response to CXCL9 **A.** the adhesion abilty of the CD133+ cells was tested by cck8. **B, C.** the CCK8 results exhibited that CXCL9 suppressed the cell adhesion through CXCR3-A, not CXCR3-B. **P < 0.01,*P < 0.05. Data are represented as mean +/− SEM.

Cell migration also involves sequential adhesion and detachment steps, chemokines and their receptors participate in the tumor cell adhesion [25]. In our research, the CD133+ Huh7, Hep-3B and PLC/PRF/5 cells were each cultured with different concentration of CXCL9 for 24h, then added to the 96-well plates, The CXCL9 100ng/ml groups showed the strongest inhibition of the cell adhesion for CD133+ PLC/PRF/5, Hep-3B, but for CD133+ Huh7 cells, the CXCL9 50 ng/ml and 100 ng/ml groups were both suppressed the most (Figure 4A). The CD133+ PLC/PRF/5 cells that receptor CXCR3-A were knockdowned by siRNA own stronger adhesion ablity than the CXCR3-B knockdown group and the control group, The CD133+ Huh7 and Hep-3B stably expressing CXCR3-A show lower adhesion ability than the control group and the stably expressing CXCR3-B group (Figure 4B and 4C). However, whether CXCR3-B was knockdowned in CD133+ PLC/PRF/5 cells or high expressed in CD133+ Huh7 and Hep-3B cells, their adhesion ability were all inhibited, this result may illustrate that CXCR3-B could act on cell adhesion not in response to CXCL9 (Figure 4B and 4C).

### CXCR3-A receptor upregulates the cell invasion through p-ERK1/2-MMP2/MMP9 signaling pathway induced by chemokine CXCL9

**Figure 5:**
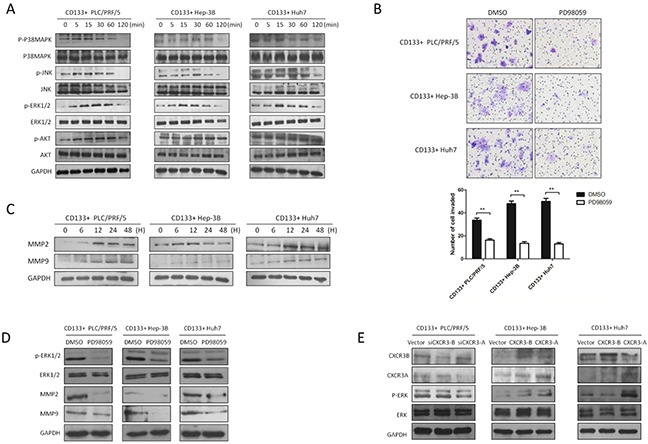
CXCR3-A receptor upregulates the cell invasion through p-ERK1/2-MMP2/MMP9 signaling pathway induced by chemokine CXCL9 **A.** P38MAPK, ERK1/2, AKT, JNK were analyzed by western blot with CXCL9 100ng/ml stimulation. **B.** Transwell assays were performed with or without p-ERK1/2 inhibitor PD98059 pretreatment, the metastasis of CD133+ liver cancer cells were inhibited significantly.**P < 0.01. Data are represented as mean +/− SEM. **C.** and **D.** MMP2 and MMP9 were increased with CXCL9 added in and regulated by p-ERK1/2. **E.** CXCL9 upregulated the p-ERK1/2 level through CXCR3-A.

To illustrate how the chemokine CXCL9 and its receptor influence the cell invasion, we screen the MAPK signaling which is of great importance on tumor metastasis, including the four main pathway ERK1/2, AKT, P38MAPK, JNK. According to the previous research in this article, CXCL9 at the concentration of 100ng/ml promoted the cell invasion and migration most. It was found that CXCL9 at 100ng/ml could significantly upregulate the phosphorylation of ERK1/2 at 15min (Figure 5A). When the phosphorylation of ERK1/2 was inhibited by its antagonist PD98059, it was found that the CD133+ PLC/PRF/5, Hep-3B and Huh7 cells invasion was reduced obviously through transwell assay (Figure 5B). As we know, matrix metalloproteinase is necessary molecular during the tumor metastasis, particularly the MMP2 and MMP9. Meanwhile the MMP2 and MMP9 could be the downstream of p-ERK1/2. So we explored the potential relationship between them. And the western blot results showed that chemokine CXCL9 at 100ng/ml could cause MMP2 and MMP9 to obvious increase in the CD133+ PLC/PRF/5, Hep-3B and Huh7 cells at 12h (Figure 5C), In the next step, we discovered p-ERK1/2 could regulate the MMP2 and MMP9 expression (Figure 5D). At last, we identified that CXCR3-A receptor isoform upregulated the p-ERK1/2 level. But in CD133+ Huh7 cells with stable expression of CXCR3-B the p-ERK1/2 level could be modestly downregulated (Figure 5E).

## DISCUSSION

Hepatocellular carcinoma (HCC) could easily cause early metastasis, which was one of the main causes of death [26]. Tumor metastasis is a complicated process, and chemokines and their receptors, which are one part of tumor microenvironment, are closely related to tumor metastasis [5, 27]. In this article, we focused on the chemokine receptor CXCR3 and its ligand CXCL9. CXCR3, which belong to the G protein-coupled seven transmembrane family of receptors(GPCR), is involved in some signaling pathways such as PI3K and MAPK [28, 29], and these pathways play a key role in tumor development [30, 31]. In our previous study, it was identified chemokine CXCL9 that it could be upregulated by HBV protein X (HBx) through activating the nuclear factor-kappa B (NF-kB) [8] and was able to promote the invasion of liver cancer cells [32]. In this study, we found that CXCR3 was upregulated in HCC tissues and was positively correlated with the metastatic potentials. However, CXCR3 has three isoforms: CXCR3-A, CXCR3-B and CXCR3-alt, but only CXCR3-A and CXCR3-B could be bound by CXCL9 [13]. The two primary ones, CXCR3-A and CXCR3-B, were reported to generate opposite physiological functions, which drew more research attention [33]. Following further research, we confirmed that CXCR3-A bound by CXCL9 promoted the HCC cells invasion and metastasis. Meanwhile, the MAPK pathways were screened and the ERK1/2 was found to be involved with the CXCR3-A and CXCL9 induced enhanced invasion of liver cancer cells. But the failure of the research in HCC tissues is that CXCL9 expression was not tested and analyzed.

The theory of cancer stem cells (CSCs) caught the interest of more and more researchers, the CSCs were defined as a group of cells having the ability to initiate tumor growth, self-renew and differentiate, CD133 had been assessed as an important liver cancer stem cells mark [21, 22, 34]. And CD133+ cancer cells were reported to be related with the chemokine CCL5 [24], so we also attempted to combine CXCL9 with the CD133+ cancer cells. Fortunately, we proved that CXCL9 could promote the invasion and migration of CD133+ liver cancer cells.

Matrix metalloproteinases (MMPs), the intriguing family of extracellular proteinases, has been implicated in various processes of developmental biology and generated widespread attention [35]. MMPs were also found to be closely related to the stem cell niche and the regulation of cancer stem cells [36]. Also the MMPs could be activated by MAPK signaling which is involved with the CXCL9/CXCR3 axis, so in this study, we wondered whether MMPs might be the downstream of CXCL9/CXCR3-A/p-ERK1/2 axis. This hypothesis was confirmed by our work.

Cell adhesion is an important step of tumor metastasis. In this research, although the results had exhibited that the CD133+ liver cancer cell adhesion ability was suppressed after co-cultured with CXCL9 for 24h through binding with CXCR3-A, the mechanism of it was not clarified. Also, the cause of why no matter CXCR3-B was knockdown or overexpressed, the adhesion ability of CD133+ liver cancer cells was both reduced.

In summary, we provided the evidence that CXCL9 binds to its receptor CXCR3-A to promote the metastasis of CD133+ liver cancer cells. In HBV-related HCC, the axis of HBx- (NF-kB)-(CXCL9/CXCR3-A)-pERK1/2-MMP2/MMP9 may play an important role in the invasion and migration of CD133+ liver cancer cells. Our findings have enriched the knowledge of the relationship between underlying HCC development and progression and the tumor microenvironment (chemokine), and also provided a potential therapeutic target for HCC.

## MATERIALS AND METHODS

### Clinical HCC specimens

All HCC specimens were obtained from surgical resection specimens of HCC patients in Tongji Hospital of Huazhong University of Science and Technology. The study was approved by the Tongji Hospital Ethics Committee and informed consent was obtained from each patient in accordance with the ethical standards of the World Medical Association Declaration of Helsinki. All the 89 pairs of tumor and matched non-tumor liver tissues were tested by PCR for CXCR3. Moreover, 40 pairs were selected for WB and IHC randomly.

### Cell culture and fluorescence-activated cell sorting

The human HCC cell lines Hep3B, PLC/PRF/5, Huh7 were purchased from the cell bank of Chinese Academy of Sciences (Shanghai, China), and routinely cultured in DMEM medium containing 10% fetal calf serum (Invitrogen Gibco, Carlsbad, CA, USA) and incubated in a 5% CO2 incubator at 37°C. Cells were trypsinized and collected, then washed two times with PBS-BSA buffer and incubated with anti-CD133 monoclonal antibody conjugated with fluorescein isothiocyanate (FITC, BD, San Jose, CA, USA) at 37°C for 30 minutes out of light. After washing three times, CD133+ cells were isolated from fluorescence-activated cell sorting (FACS) Calibur (BD, San Jose, CA, USA).

### Quantitative polymerase chain reaction (qPCR)

Total RNA was extracted using Trizol reagent (Invitrogen, Carlsbad, CA, USA). Reverse-transcribed complementary DNA was synthesized using the PrimeScript RT reagent kit (TaKaRa, Otsu, Japan). Real-time polymerase chain reaction was performed using SYBR Premix ExTaq (TaKaRa, Otsu, Japan) on an ABI Step One Real-Time PCR System (Applied Biosystem, Carlsbad, CA, USA). The amount of mRNA for each gene was normalized by GAPDH, and the relative expression levels were calculated using the 2^−ΔΔCt^ method. Primers were designed using NCBI Primer-BLAST. CXCR3 primer 5′ TCCACCTAGCTGTAGCAGACAC, 3′ primer TCCTGCGTAGAAGTTGATGTTG; CXCR3-A primer 5′ CCAAGTGC-TAAATGACGCCG; CXCR3-A 3′ primer: CAAAGGCCACCACGACCACCACCA which yield products of 770 bp; CXCR3-B 5′ primers ATGGAGTTGAGGAAGTACGGCCCTGGAAG; CXC R3-B 3′ primers: AAGTTGATGTTGAAGAGGGCAC CTGCCAC, which yield 545-bp products [37].

### RNA interference and overexpression

Transfection was performed using Lipofectamine 2000 (Life technologies) following the standard method. The siRNA sequences (Ribo Company, Guangzhou, China) used are as follow: CXCR3-A 5′ GAACUUCAGCUCUUCCUAU dTdT, 3′ dTdT CUUGAAGUCGAGAAGGAUA; CXCR3-B 5′ GGAGC UGCUCAGAGUAAAU dTdT, 3′ dTdT CCUCGACGAGUCUCAUUUA. For establishment of stable expressing cells, plasmids (Genechem Company, China) were transfected into cells with lipofectamine 2000 manufacturer's instructions. We got the stable overexpression transfectant by adding G418 (Sigma-Aldrich) for 4 weeks.

### Western blot

Blots in PVDF membranes were incubated overnight with primary antibody at 4°C. AKT (1:1000), p-AKT (1:1000), ERK1/2 (1:1000), p-ERK 1/2 (1:1000), SAPK/JNK(1:1000), p-SAPK/JNK(1:1000), p-P38MAPK(1:1000) (Signalway Antibody LLC, Maryland, USA), CXCR3 (1:600, ABGENT Company, San Diego, USA), CXCR3-A and CXCR3-B (1:500, BOSTER Company, Wuhan, China), followed by anti-rabbit IgG (1:3,000; Sigma, CA) for 1 h at room temperature, and the signals were detected with an ECL assay kit (Amersham, Buckinghamshire, UK).

### Immunohistochemistry and immunofluorescence staining

Tissue sample slides were deparaffinized with dimethylbenzene, followed by gradient alcohol dehydration, and incubation with 3% hydrogen peroxide. Primary anti-CXCR3 antibody (1:400, R&D, Minneapolis, USA) was incubated with the slides overnight at 4°C, then the secondary antibody was added for 30 minutes at room temperature, then the slides were visualized with peroxidase-labeled streptavidin-complexes/DAB, and counterstained with hematoxylin, mounted and visualized under an inverted microscope. Immunofluorescence staining was performed as previously described [38].

### Cell migration and invasion assay

Wound-healing was performed as described previously [39]. Matrigel-coated invasion inserts (BD Biosciences, NJ, US) with 8 μm-pored membranes were used for invasion assays. The upper chamber was seeded 3 × 10^4^ cells in 100 μl DMEM medium without serum. The lower chamber was added to the 600 μl DMEM medium containing different concentrations of chemokine CXCL9. For this assay, cells on the upper chamber were removed with a cotton swab, and cells on the lower chamber were fixed and stained. Three random fields were chosen to take pictures and count.

### In vivo metastasis model

All experimental procedures involving animals were performed in accordance with the Guide for the Care and Use of Laboratory Animals (National Institutes of Health publication nos. 80-23, revised 1996). 1×10^6^ cells were injected into the tail vein. Mice in tail vein injection groups were sacrificed on day 60 and their livers were dissected, fixed in 10% buffered formalin, and prepared for histological analysis.

### Cell adhesion

96-well plates were coated with 10 μg/mL fibronectin in PBS at 4°C overnight, and then each well was incubated with 200 μl of heat-denatured 10 mg/mL BSA in PBS at 37°C for at least 1 h. After washing the wells twice, each well was seeded 3 × 10^4^ cells in 100μl PBS, and then incubated for 60 min in a 5% CO2 incubator at 37°C. Wash the wells three times with PBS, then incubated with Cell Counting Kit-8 (CCK8) reagent for one hour; finally, the OD value was visualized on multimode reader.

### Statistical analyses

The data are presented as mean ± SEM and all experiments were performed in triplicate if not specified. CXCR3 expression was compared with demographic and biological parameters by cross-table analysis performed using a Fisher exact Chi square test. Statistical comparisons between groups were made by the student's t test. Trials which p values are less than 0.05 are considered significant.
